# Perception of malaria risk in a setting of reduced malaria transmission: a qualitative study in Zanzibar

**DOI:** 10.1186/1475-2875-12-75

**Published:** 2013-02-22

**Authors:** Julie A Bauch, Jessica J Gu, Mwinyi Msellem, Andreas Mårtensson, Abdullah S Ali, Roly Gosling, Kimberly A Baltzell

**Affiliations:** 1 Department of Global Health Sciences, University of California San Francisco, 50 Beale Street, Suite 1200, San Francisco, CA, 94105, USA; 2 Zanzibar Malaria Control Programme, Zanzibar Ministry of Health, PO Box 503, Zanzibar, Tanzania; 3 Department of Medicine Solna, Malaria Research, Retzius väg 10, Karolinska Institutet, 171 77, Stockholm, Sweden; 4 Department of Public Health Sciences, Division of Global Health (IHCAR), Nobels väg 9, Karolinska Institutet, 171 77, Stockholm, Sweden; 5 Malaria Elimination Initiative, Global Health Group, University of California San Francisco, 50 Beale Street, Suite 1200, San Francisco, CA, 94105, USA; 6 Department of Family Health Care Nursing, University of California San Francisco, 2 Koret Way, #431M, San Francisco, CA, 94143, USA

**Keywords:** Perception of risk, Malaria, Zanzibar, Qualitative, Focus group

## Abstract

**Background:**

Malaria transmission has declined dramatically in Zanzibar in recent years. Continuing use of preventive measures such as long-lasting insecticidal-treated nets (LLINs), and use of malaria rapid diagnostic tests (RDTs) are essential to prevent malaria resurgence. This study employed qualitative methods to explore community perceptions of malaria risk and adherence to prevention measures in two districts in Zanzibar.

**Methods:**

Key informant interviews with 24 primary health care providers and 24 focus group discussions with local residents in Zanzibar districts Wete and Central were conducted during April and May 2012 focusing on perception of malaria risk, current preventive practices used, reasons for using preventive practices and effective strategies for malaria control.

**Results:**

Health care providers and residents appear to be aware of the decreasing incidence of malaria. Both groups continue the use of malaria preventive practices in this low and seasonal transmission setting. The most important preventive measures identified were LLINs, indoor residual spraying (IRS), and education. Barriers to malaria prevention include: lack of staff at clinics, insufficient number of LLINs distributed, and inadequate malaria education. Reasons for continued use of preventive practices include: fear of malaria returning to high levels, presence of mosquitoes during rainy seasons, and concern about local cases from other villages or imported cases from mainland Tanzania. Mosques, clinics, schools and community meetings were listed as most important sources of education. However, residents express the desire for more education.

**Conclusion:**

Health care providers and residents generally reported consistent use of malaria preventive measures. However, maintaining and continuing to reduce malaria transmission will require ongoing education for both health care providers and residents to reinforce the importance of using preventive measures. Successful efforts to reduce malaria in Zanzibar will be jeopardized if residents believe that they are no longer at risk for malaria. In future studies, a year-round evaluation of the perception of malaria risk and use of preventive measures will inform the timing of education and prevention strategies for sustained malaria control.

## Background

Malaria transmission has declined significantly in Zanzibar, from 35-40% prevalence in 1995 to less than 2% in 2010 [1]. Currently Zanzibar is working towards an elimination goal on both Unguja and Pemba islands [1]. Historically, Zanzibar has come close to achieving elimination, however, due to a complex interplay of factors including the relaxation of vector control measures, malaria resurged and the last attempt at elimination was in the 1980s [1].

Importantly, Zanzibar has the characteristics of a pre-elimination country: it is at the margins of a malaria-endemic region (East Africa), transmission has been reduced and incidence is low [2]. To maintain current gains in malaria reduction, the continued use of preventive measures are necessary. In 2009 in Zanzibar, 96% of surveyed individuals identified at least one malaria preventive measure [3]. Eighty-eight percent of households own at least one mosquito net [2]. However, use of insecticide-treated nets (ITNs) or long-lasting insecticidal nets (LLINs) ranges widely across districts in Unguja and Pemba [1]. The 2010 Demographic and Health Survey showed 65.9% of children under five years of age and 64.1% of pregnant women sleep underneath an ITN [2]. These numbers indicate a three-fold increase from the 2004–05 survey [4]. In spite of recent improvements in bed net use, further studies are needed to understand reasons a third of children under five years of age and pregnant women are not using bed nets [2].

Current indoor residual spraying (IRS) coverage in Zanzibar is high with over 85% of targeted households receiving IRS treatment [5]. Recently the strategy for IRS use in Zanzibar has changed from universal coverage to targeted spraying. However, a study on IRS use in rural villages of Mozambique found many residents refused to participate in IRS when malaria transmission was perceived to be low. Additionally, the purpose of IRS was unclear to both accepting and non-accepting households [6].

Social and cultural factors, although less directly studied, are also important determinants to net usage and adherence to other preventive behaviour. Men are not targeted for interventions although in some contexts they are at elevated risk for malaria. For instance, men in Vanuatu and Solomon Islands are more active outdoors during evening hours, which puts them at risk [7,8]. Additionally, financial constraints are cited as a limitation for net ownership [9,10].

Decision-making around malaria prevention involves multiple sources of input [11,12]. In Zanzibar, health care and health education is available through public and private health facilities, local privately owned dispensaries, and traditional healers [12]. To ensure compliance with malaria prevention strategies, it is necessary to understand how and where residents are receiving information on this topic.

As the burden of malaria decreases in Zanzibar, it will be increasingly important to understand residents’ perception of continuing preventive measures to avoid “elimination fatigue” [1].

The aim of this study was to describe community and health care providers perception of malaria risk, reasons for adherence and non-adherence to malaria preventive practices and to identify effective communication strategies and educational information used to encourage malaria prevention in a setting of low malaria transmission.

## Methods

### Study population and study sites

This qualitative study was conducted in two districts in Zanzibar. The study population for both the focus groups and key informant interviews was drawn from six villages and primary health care facilities in each Wete and Central districts on Pemba and Unguja islands, respectively. Malaria prevalence in 2008 was 0.1-0.2% in Central District and 0.6-2.5% in Wete [1]. These districts were chosen based on a recent increase in reported malaria cases [13]. These districts are also considered representative of their respective islands based on a recent modest increase of malaria prevalence. A total of 12 villages and 12 primary health care facilities were included in the study. Villages in Central District included; Uzini, Mwera, Pongwe, Tunguu, Charawe, and Umbuji. Villages in Wete District included; Mzambarauni, Pandani, Ukunjwi, Kiuyu Minungwini, Kangagani, and Ole.

Key informants were identified in collaboration with Zanzibar Malaria Control Programme (ZMCP) and district health officers. The study team visited identified facilities and briefly explained the purpose and objectives of the study. Key informants included clinical officers, nurses and medical assistants to capture the range of health care providers interacting with patients.

Focus group discussions were conducted with men and women in villages adjacent to the primary health care facilities. Eligible participants for focus groups included residents in Central or Wete Districts, aged 18 years and older, and willing to provide informed consent for participation. Researchers verified eligibility and chose participants to represent different demographics in the village.

### Procedures

The study used a semi-structured interview guide for key informant interviews and focus group discussions. Additionally, observation of malaria prevention messages posted in primary health care facilities and surrounding villages was carried out to assess information given to local residents about malaria.

### Data collection

Interviews and focus group discussions were conducted during a six-week period in the rainy season, April to May 2012, which historically coincides with the yearly peak malaria transmission period in Zanzibar.

Focus group discussions explored residents’ perceptions of malaria risk, preventive practices used (i e, bed net use, IRS, and biomedical treatment of febrile illness), factors influencing adherence (i e, persons who influence health-seeking behaviour, effects of health messages and campaigns), and health communication strategies.

The key informant interviews explored health care providers’ perceptions of malaria risk, current preventive practices used by residents from the surrounding villages, current resident perceptions of malaria risk, and efficacy of health messages in the communities. Perspectives from health care providers were compared to that of residents in the community for congruency.

Focus group discussions were conducted in the villages at a location and time convenient for the residents. Focus groups were separated by gender. Key informant interviews were conducted in a private setting at primary health care facilities. Interviews and discussions ranged from 30 to 60 minutes. Each interview or discussion was conducted in Kiswahili by a translator and by a researcher. The interviews were audio recorded, and handwritten notes were taken to supplement recordings.

Observations of clinic equipment and educational posters were made in primary health care facilities and in the village environments to provide context to the qualitative information gathered in key informant interviews and focus groups. These observations included informal conversations with the health care providers and site tours.

### Data analysis

The audio recordings were transcribed and translated to English by a bilingual Kiswahili-English Ministry of Health staff member. Data were imported into Dedoose^TM^ qualitative analysis software. Open *in vivo* coding, based on thematic content analysis was carried out by two researchers to identify major themes. Themes were compared and consensus was reached for each theme.

### Ethical considerations

This study was approved by the Zanzibar Medical Research and Ethics Committee and the University of California, San Francisco Committee on Human Research. All participants signed a written consent prior to participation in the study. No personal identifiers were recorded or transcribed. Participants were not paid in this study.

## Results

Interviews were conducted with 24 practicing health care providers and 24 focus groups were conducted with 134 residents of 12 villages in Central and Wete Districts.

Key informants included clinical officers, public health nurses, staff nurses, health orderlies and medical assistants. To maintain cultural sensitivity, focus groups were divided between men and women in each village. All participants were between the ages of 18 and 72.

The following major themes were identified (Table 1):

Theme 1 Health care providers and residents believe they are at risk for malaria, but expressed numerous barriers to care for protecting themselves

Health care providers and residents reported that RDTs and ACTs are consistently available at primary health care facilities. However, health care providers listed barriers to care for their patients including lack of staff, and lack of laboratory equipment.

*“Challenges. We have many of them. Our centre is big but we have very few staff. There is a lot of equipment in the lab we don’t have.”* (HCP 1)

[Author’s note: HCP indicates Health Care Provider]

Residents stated an understanding of the importance of environmental sanitation for malaria prevention. They actively eliminate mosquito-breeding sites around houses, trees, and by removing standing water. However, they expressed a lack of equipment for cleaning the environment.

*“The tools have decreased…the equipment to work, like rakes…”* (R 10 - M)

[Author’s note: R indicates Resident, M indicates Male, F indicates Female]

*“There are a lot of challenges. Some understanding the importance, but others don’t want to understand. You will tell them ‘in this ditch there is dirty water and it’s dangerous. Clean your environment.’ But in a week you follow up and it’s like they didn’t understand what you meant. And this is a challenge.”* (HCP 25)

Residents and health care providers reported the existence of village health committees. The health committees consist of village members and local health care providers. Residents stated that inactive and under-informed health committees prevent them from receiving better information on malaria prevention methods. Long-lasting insecticide treated nets were distributed throughout Zanzibar in March 2012. Rumours circulated that the new nets had caused serious side effects. Residents expressed that better informed and more active village health committees can improve information dissemination.

*“People have been complaining…We haven’t been given any instructions about using nets. Only after the effects showed were we told to wash the nets…others said to leave it outside for a time…”* (R 12 - F)

*“The way to get rid of malaria completely is to increase the services that we have right now, to make them better. The health committee in the village needs to be given resources to move forward, they need to get more education to improve those who are giving education.”* (R 9 - M)

Health care providers express the desire to educate patients on malaria, but express time restraints as a barrier. Health care providers also expressed that they need more frequent training on malaria in order to better teach the local community.

*“I see it’s not good. For now it’s really difficult. You yourself, you have to be the doctor, give the shots, give the medicine, take care of the women the children, and give counselling for family planning.”* (HCP 12)

*“The easiest way is for us to get education, to get refresher course… to give us the education then we can give it to the people in the community.”* (HCP 12)

Theme 2 Residents believe education is critical in malaria prevention. Mosques, clinics, schools and community meetings provide education. However, residents consistently express desire for more education

Zanzibar residents and health care providers discussed the importance of multiple strategies in disseminating health education. Current education strategies include the use of mosques, clinics, schools and community meetings to distribute health information. Residents have also heard malaria health messages through radio or television. However, the education methods most preferred by residents are group meetings or seminars.

*“These meetings are the best ways…because education will reach faster…those who come here [clinic] are those who are sick. We need to reach even those who are not sick, therefore doing meetings outside are better.”* (HCP 14)

*“… we need all kinds of education because education will take us out of the darkness.”* (R 3 - M)

Residents and health care providers differed on their opinion on the effectiveness of mass media campaigns. Some residents described radio, television and posters as important health messaging routes, while others preferred theatre presentations.

*“To bring it [education] through TV is easy to spread to those who go watch…and there are those who don’t know how to read… There can be education through the radio, but some don’t listen to the radio.”* (R 9 - F)

*“All three ways are good but it is on each person’s time. I can read the newspaper, you read, you watch TV and I don’t, that person listens to the radio and another doesn’t listen. It is important to have education available through village meetings so that we can get the training. Although there will be some cost to it…but if you give a person something of quality, he will take it and it will stay in his head.”* (R 4 - M)

*“A good way - I have seen people understand - is to do theatre…if a person has fever what is it like, what should he do, so that they will know that it is dangerous. If you show with theatre they will understand very much…role play will teach a lot.”* (HCP 17)

Residents expressed a desire for education before net distribution or IRS distribution campaigns in order to improve understanding and use of prevention methods.

*“Just health education, then net…. health education about malaria…health education first and then the nets.”* (HCP 1)

*“Bring the malaria education, very few got it the day they did spraying. Some shehias (*Author note: *shehia* is the general name for a village in Zanzibar*) had education…that came with the spraying…to finish this issue completely, they who have the purpose of giving education, they should come to remind us.”* (R 6 - F)

School-based health education is expressed as an important way to educate children about malaria. As fewer children are experiencing malaria in Zanzibar, there is concern that children may not have the memory of malaria to continue use of preventive methods. Health care providers and residents emphasize the need to educate children about malaria.

*“Children are supervised by their parents, so father and mother should use prevention strategies and explain to children the danger of the disease and to make sure that they stay in the net to avoid mosquito bite…so by the time they have grown they have received education. Maybe in school also, school children can get education…to prevent malaria.”* (HCP 19)

Zanzibar residents receive malaria prevention education periodically, but are concerned if they do not receive education frequently they will forget. As all participants in this study were over the age of 18, they have experienced the rise and fall of malaria transmission in Zanzibar in recent years.

*“First is education…the most basic thing is to give education once in a while, so that it can continue…because today you give education and after a year you have to continue with education again and again.”* (HCP 13)

According to the study participants, without education to the community, acceptance and use of prevention methods may decline.

*“People are not getting enough education, which leads to people not agreeing to have their houses sprayed, this is a lack of education…a lack of education is related to malaria being present.”* (R 12 - F)

*“We need to educate people to understand that malaria is still present. Not to take the idea that there is no malaria. Even if you test someone negative, if they say there isn’t anything it means they already forgot. Therefore we need to tell them that malaria is still present, and we need to educate our patients…to continue to prevent malaria, to cover ourselves with our nets, to have anyone with fever come to the hospital to get tested.”* (HCP 4)

In areas where the health committee is not active, residents request more health committee involvement. In addition to radio, television, and posters, residents also feel that health committees are important avenues for malaria education. However, residents expressed concern that information provided to health committee members was not being delivered to the community.

*“A good way is to come together like this, to have meetings or group seminars.”* (R 9 - M)

*“I agree with shehia that education is not enough…it is the leaders of that committee who are often getting the education and not the community… Now I am saying that it is the leaders of the committee who are getting the education, and each leader is understanding on a different level. …the leaders are important to get education because they are elected from the people. But in addition to this the education must reach the community.”* (R 5 - M)

Theme 3 Residents are aware of decreasing malaria transmission. However, there is fear that people can get malaria, and that malaria transmission will rise if preventive measures are not used

Residents believe that malaria levels used to be high five years ago, and much higher 10 years ago, but are now much lower. They attributed some of the decline to use of bed nets, better anti-malarial medications, and IRS. However, some residents expressed concern that immunity to malaria has also declined, leaving them at greater risk of malaria should it resurge.

*“Malaria, to say the truth, has decreased by 90%, since using nets.”* (R 8 - M)

*“It was high before, not so high a few years back, but 10 years ago.”* (R 3 - F)

*“From 10 years ago it has gone down.. from five years ago, somewhat.”* (HCP 4)

Even though residents and health care providers believe malaria is lower now, they think that it is still possible to get malaria.

*“It is possible to get [malaria] because we have no certainty that malaria is completely gone.”* (HCP 18)

Significant fear persists for getting malaria and for malaria levels to rise again. Although malaria cases are less frequent, residents and health care providers are aware when they occur in a community. Residents understand that every malaria case presents the possibility of getting malaria and the potential rise in malaria transmission again.

*“We’re afraid because it has killed. Malaria has killed a lot.”* (R 10 - F)

“*Myself I am afraid. Even though there is no malaria like other years, I am afraid even a little because now we have no immunity.”* (HCP 19)

*“We fear malaria because it will rise up again to how it was before.”* (HCP 21)

*“We know that this problem is decreasing and is leaving, but it is possible that it will come up again. Therefore, it is important to stay in the condition to be afraid and to use nets.”* (HCP 20)

Health care providers and residents believe travellers pose a threat for continued malaria transmission. Residents and health care providers were asked to describe the differences in malaria transmission between Zanzibar and mainland Tanzania. Both residents and health care providers perceive that malaria transmission is higher in mainland Tanzania than in Zanzibar. There is concern that travellers from mainland Tanzania are bringing malaria to Zanzibar. Within Zanzibar, there is concern that villages with higher reported cases of malaria incidence may spread malaria to other villages.

*“There are a lot of things, maybe transfer in transfer out maybe because our neighbour has no project like us. Maybe from Tanga mainland…we have a lot of fishers…so malaria comes. Other travellers may transfer in the same country to and from Zanzibar, maybe Dar es Salaam. Also malaria is not finished in Pemba, still we have but in low amounts. If one stays in Wete and then goes to rural areas with malaria, so malaria in the rural area will remain, this is the problem also.”* (HCP 19)

*“They are afraid that it will come again, because there are other shehias that still have malaria.”* (HCP 24)

There are differing opinions on whether travellers use bed nets and other prevention methods while travelling. Some travellers are reported to carry nets with them when travelling. Residents suggested possible testing for those entering Zanzibar to ensure that infection is not being imported from elsewhere.

*“Because people are coming and going every day now, maybe people don’t use nets so malaria will come again I think.”* (HCP 19)

*“We need to enter from one way only and we need to get tested whether we like it or not. The government must put this on its agenda because we can finish malaria. …. I think it will be an easy way to ensure that someone has come but they don’t have malaria.”* (R 4 - M)

Theme 4 People currently use malaria preventive methods, and perceive that they are useful to prevent malaria

Residents and health care providers were asked to describe current prevention methods that they use to prevent malaria. Residents state that using bed nets, receiving IRS every year, and doing environmental sanitation have worked in reducing malaria transmission.

*“A good way is to use nets, to agree to spraying, to clean the environment and get rid of standing water to take away mosquito breeding sites.”* (HCP 23)

*“It is possible for malaria to happen, we believe in nets, education, sanitation, that which we have been hearing. But if we were to cover our ears, malaria will come. If we do not use nets, don’t clean…mosquitoes will breed.”* (R 3 - F)

However, they state that the number of nets distributed is insufficient. The maximum number of nets distributed per family is three. Most families are large and cannot place each family member under a distributed net. Therefore, parents choose to place children under the nets and the adults sleep without nets.

*“We need to get more nets because in the family, some even use kangas (*Author note: *kanga* is a cloth used for clothing and to carry infants currently costing approximately $1 USD*) as nets to cover themselves. We need to distribute to all.”* (HCP 7*)*

*“We have received nets but not enough because for families of five or four they got two [nets], families of 10 they got three, now you find others that haven’t received them.”* (R 2 - M)

*“The children sleep under the nets, father and mother sleep outside of the net.”* (R 10 - M)

Additionally, residents express that purchasing a net is unrealistic for their budget. Cost for an ITN or LLIN in Zanzibar is currently approximately $4-6 USD. Large families with many children state that they cannot afford to purchase enough nets for the entire family.

*“Some will buy…but you need to have the ability to buy a net. It requires the ability to buy for 12 children, it is not something easy.”* (R 9 - M)

Residents have heard rumours that the distributed nets were harmful. However, through conversations with village leaders and radio announcements, these rumours have been dispelled and residents believe that nets continue to be useful in malaria prevention. Residents suggest that the rumours were started by private bed net companies to sway people to purchase nets instead of using the free, distributed nets.

*“People died, people felt sick, people were itching, but the Ministry of Health sat together and found the answer that this rumour was disproven because in every shehia there was not a single report from a hospital in Zanzibar or in Pemba that anyone had gotten side effects from the nets.”* (R 4 - M)

*“We haven’t heard of it, we only heard rumours but we don’t know if it is for business or what…”* (R 7 - F)

When specifically asked about heat-related issues and net use, residents express that increased heat plays a role in inconsistent use of nets. Furthermore, residents report inconsistent use of nets in the dry season when there are fewer mosquitoes.

*“A big percent of people are not using, because it causes heat.”* (HCP 24)

*“We use it…when there are mosquitoes we use it, maybe unless it is hot.”* (R 8 - F)

*“If mosquitoes are not present, to say the truth we don’t like to use the nets because of the heat. But when mosquitoes are around we do our best with it.”* (R 8 - F)

Finally, residents believe that IRS contributes to the control of malaria. Zanzibar now uses targeted spraying in hotspot areas. A hotspot is defined as an area experiencing a two-fold or higher increase in malaria incidence from the previous week (per communication with Zanzibar Malaria Control Programme). However, few residents understand the purpose of targeted spraying in selected villages, and are concerned about malaria transmission in their village. In non-hotspot areas, residents are requesting that IRS remains consistent throughout the villages. Furthermore, residents feel that spraying more than once a year is necessary for the continued control of malaria.

“This time the work only went to some of the shehias and still now we have not received it, because if you say to spray one shehia and not another, Anopheles is here and is there, but if we plan programmes we want to spray the whole of Zanzibar, this will help.”

(R 2 - M)

*“… increase the efforts of IRS, and to use the spray more often to get rid of [malaria] entirely. It should not be that they think malaria is low and then they stop. Therefore there needs to be more education, and more spraying.”* (R 9 - M)

*“…We should increase spraying, spraying is done every six months meaning if we do it every three months we will get rid of malaria entirely.”* (R 6 - M)

**Table 1:** Major result themes

**Theme 1**	Health care providers and residents believe they are at risk of malaria,but expressed numerous barriers to care for protecting themselves.
**Theme 2**	Residents believe education is critical in malaria prevention. Mosques, clinics, schools and community meetings provide education. However, residents express the desire for more education.
**Theme 3**	Residents are aware of decreasing malaria transmission. However, there is fear that people can get malaria, and that malaria rates will rise if preventive measures are not used.
**Theme 4**	People currently use malaria preventive methods, and perceive that they are useful to prevent malaria.

## Discussion

This qualitative study in Zanzibar examined the perception of current malaria risk in health care providers and residents who visit government primary health care facilities. Overall, both health care providers and residents believe that malaria transmission has decreased dramatically over the past 10 years. Importantly, both groups also believe that although transmission rates are lower, they are still at risk for contracting malaria. The fear of malaria and having transmission rates rise again drives residents to continue with preventive measures. These findings, plus four additional findings from this study could be useful in other settings where an improved understanding of malaria risk perception is needed to guide pre-elimination measures.

First, interviews with health care providers and residents reveal that public health facilities are perceived to be consistently stocked with RDTs and ACT free-of-charge. This finding is important as self-treatment without testing (via purchasing medication at private dispensaries) may contribute to development and spread of anti-malarial drug resistance [12].

The majority of health care providers and residents report that public health care facilities are understaffed. Understaffed facilities can result in inadequate care given to residents. Health care providers interviewed feel that additional staff would benefit the health care facilities. Currently, staff members fulfil multiple roles in the health care facility, limiting the volume of patients seen and the quality of care given. Residents have long wait times at government health care facilities to see a health care provider, and often are not seen the day they arrive. Health care providers feel they do not have enough time to see all patients who present to the health care facilities. If there is not adequate time to see and teach each patient, the possibility of a patient disbelieving a negative malaria test increases. Lack of trust in malaria testing results may lead to self-treatment, increasing the risk for drug resistance, improper treatment of the true underlying disease and under reporting of true malaria cases.

Second, it is important that residents are continuing to receive malaria education that encourages the use of malaria prevention practices. Residents interviewed for this study are concerned that reducing education may lead to a reduction in the use of prevention methods. Health messaging and education from existing community gatherings were cited most often as the preferred educational strategies. For example, mosques are frequent gathering places in this predominantly Muslim community. Health messaging through mosque loudspeakers and involving religious teachers in delivering health education was suggested by residents as an effective education technique.

Community meetings are routinely used to convey information in Zanzibar villages; therefore, taking advantage of this existing infrastructure may be an efficient way to deliver health messages. In this study, residents frequently cited community meetings led by health committees as the preferred method of receiving health education. Health committees exist in every village, where the village leader (*Sheha*) and local health care providers convene. Under the direction of Shehas and health care providers, correct health messages may reach a greater number of village residents. Education during net distribution campaigns strengthens the message of proper net use and proper net hanging. Active and informed health committees may be instrumental in dispelling rumours, such as bed nets are dangerous. Residents consistently suggest improving the quality of education that they received. Therefore, it is important to ensure that health care providers and health committee members are informed regularly on local malaria epidemiology and updated on preventive measures used.

Inserting malaria education into school curricula may be useful in this setting of low malaria transmission. Due to decreased malaria transmission rates, many Zanzibari children have not experienced malarial illness. Residents suggest that the best way to teach children to use malaria preventive measures is through formal health education at school.

Third, residents understand that malaria transmission rates are low, but still fear getting malaria. Harnessing the current ‘malaria-is-still-present’ perception is vital to strengthen adherence to prevention practices. Residents remember the history of malaria resurgence in Zanzibar and believe that relaxing preventive measures may lead to resurgence. Now that malaria transmission is reduced, it is important to remind residents that consistent use of preventive measures may ensure the past does not repeat itself. In general, residents and health care providers feel that the risk of malaria is higher in mainland Tanzania than in Zanzibar. Residents and health care providers have expressed concern about travellers bringing malaria from mainland Tanzania. Continued research on malaria transmission patterns is important to guide elimination efforts. Furthermore, malaria preventive measures are inconsistent for those who travel, according to study participants. Stressing the importance of travelling with personal nets is important to consider for malaria control on Zanzibar. Screening travellers in Zanzibar may be necessary in the future to avoid imported infections.

Finally, despite a recent net distribution in Zanzibar, residents consistently expressed that insufficient numbers of nets were distributed to large households. As reported above, residents find kangas more economical than LLINs, however, there seems to be a misunderstanding regarding the lack of protection provided by kangas. Additionally, as the children are primarily placed under the nets, adults are often left outside of nets and at risk for mosquito bites. Future net distributions should include universal coverage for all household members. Decreased immunity in adults will take on more public health significance as malaria transmission decreases, as adults will suffer from more serious disease when infected.

The issue of IRS was controversial among the study participants. Most residents in this study felt spraying an entire village is more effective than targeted IRS, the current strategy in Zanzibar. Island-wide IRS campaigns have now changed to become targeted IRS in villages where malaria rates have increased. As a result, some residents are concerned that malaria can increase in areas that do not receive IRS. Residents express a desire for consistent IRS in every village, instead of waiting for malaria to increase in order to receive IRS. With limited resources for malaria control, Zanzibar has chosen to move forward with targeted IRS. Therefore, information about targeted IRS and the implications of this strategy need to be clarified and disseminated to prevent misconceptions of the absence of IRS in selected villages.

Interestingly, men in focus groups often mentioned the issue of spending time outside after dusk. It is not uncommon for men to gather during the evening and night to talk, eat, and watch television. However, they mentioned that during this time they are not protected from mosquitoes, and know that they are exposed to mosquito bites. As a solution to this problem, they have requested the use of outdoor spraying, a strategy some residents remembered from the past. Looking forward, a culturally sensitive solution that accounts for this daily practice will be important for their protection.

### Limitations

This study has some limitations. First, it was conducted during the rainy season when risk of malaria is increased. This may have had the effect of enhancing residents’ perception of the importance of using preventive measures. Second, it is possible that the attitudes and answers of the informants may have been impacted by the fact that they were chosen by the Zanzibar Malaria Control Programme. Finally, the study was conducted approximately one month after net and IRS distribution. Therefore, our results may be influenced by heightened awareness of malaria at this time. Future studies may consider exploring how risk perception changes throughout the seasons in Zanzibar.

## Conclusion

Health care providers and residents generally reported consistent use of malaria preventive measures. In Zanzibar, it appears that residents and health care providers understand the significance of using preventive measures, even in areas of very low malaria transmission. However, maintaining and continuing to reduce malaria transmission will require ongoing education for both health care providers and residents to reinforce the importance of using preventive measures. Successful efforts to reduce malaria in Zanzibar will be jeopardized if residents believe that they are no longer at risk for malaria. In future studies, a year-round evaluation of the perception of malaria risk and use of preventive measures will inform the timing of education and prevention strategies for sustained malaria control.

## Competing interests

The authors declare that they have no competing interests.

## Authors’ contributions

JB and JG conceived of the study and conducted initial data analysis. KB and RG provided supervision on all aspects of study design, data analysis and manuscript preparation. MM and AA coordinated all aspects of the study in Zanzibar and assisted with study design. All authors have read and approved the final manuscript.

## References

[B1] Zanzibar Malaria Control Program Ministry of health and social welfare Malaria elimination in Zanzibar: a feasibility assessment 2010

[B2] Feachem RG Phillips AA Hwang J Cotter C Wielgosz B Greenwood BM Sabot O Rodriguez MH Abeyasinghe R Ghebreyesus T Snow RW Shrinking the malaria map: progress and prospects Lancet 2010 376 1566 1578 10.1016/S0140-6736(10)61270-6 21035842 PMC3044848

[B3] Zanzibar Malaria Control Programme Ministry of health and social welfare Zanzibar communication strategy for malaria control 2008–2012 2009

[B4] National Bureau of Statistics (NBS) ORC macro Tanzania demographic and health survey 2004–05 2005 Dar es Salaam

[B5] Tanzania Commission for AIDS (TACAIDS), Zanzibar AIDS Commission (ZAC) National bureau of statistics (NBS), office of the chief government statistician (OCGS), macro international Inc Tanzania HIV/AIDS and malaria indicator survey 2007–08 2008 Dar es Salaam

[B6] Munguambe K Pool R Montgomery C Bavo C Nhacolo A Fiosse L Sacoor C Nhalungo D Mabunda S Macete E Alonso P What drives community adherence to indoor residual spraying (IRS) against malaria in manhica district, rural Mozambique: a qualitative study Malar J 2011 10 344 10.1186/1475-2875-10-344 22111698 PMC3339361

[B7] Atkinson J Fitzgerald L Toaliu H Taleo G Tynan A Whittaker M Riley I Vallely A Community participation for malaria elimination in tafea province, Vanuatu: part I. Maintaining motivation for prevention practices in the context of disappearing disease Malar J 2010 9 93 10.1186/1475-2875-9-93 20380748 PMC2873527

[B8] Atkinson J Bobogare A Fitzgerald L Boaz L Appleyard B Toaliu H Vallely A A qualitative study on the acceptability and preference of three types of long-lasting insecticide-treated bed nets in Solomon Islands: implications for malaria elimination Malar J 2009 8 119 10.1186/1475-2875-8-119 19497127 PMC2699345

[B9] Kinung’hi SM Mashauri F Mwanga JR Nnko SE Kaatano GM Malima R Kishamawe C Magesa S Mboera L Knowledge, attitudes and practices about malaria among communities: comparing epidemic and non-epidemic prone communities of muleba district, north-western Tanzania BMC Publ Health 2010 10 395 10.1186/1471-2458-10-395 PMC2910681 20602778

[B10] Minja H Schellenberg JA Mukasa O Nathan R Abdulla S Mponda H Tanner M Lengeler C Obrist B Introducing insecticide-treated nets in the kilombero valley, Tanzania: the relevance of local knowledge and practice for an information, education and communication (IEC) campaign Trop Med Int Health 2011 6 614 623 11555427 10.1046/j.1365-3156.2001.00755.x

[B11] Williams HA Jones C A critical review of behavioral issues related to malaria control in sub-Saharan Africa: what contributions have social scientists made? Soc Sci Med 2004 59 501 523 10.1016/j.socscimed.2003.11.010 15144761

[B12] Alilio MS Eversole H Bammek J A KAP study on malaria in Zanzibar: implications for prevention and control. A study conducted for UNICEF Sub-office Zanzibar Evaluat Program Planning 1998 21 409 413 10.1016/S0149-7189(98)00030-5

[B13] Zanzibar Malaria Control Programme Zanzibar malaria early epidemic detection system biannual report 2009 1 (No.2); March 2010

